# Quantification and Topographical Distribution of Terminal and Linked Fucose Residues in Human Spermatozoa by Using Field Emission Scanning Electron Microscopy (FE-SEM)

**DOI:** 10.3390/ijms222111947

**Published:** 2021-11-04

**Authors:** Laura Robles-Gómez, Paula Sáez-Espinosa, Eliana Marina López-Viloria, Andrea López-Botella, Jon Aizpurua, María José Gómez-Torres

**Affiliations:** 1 Departamento de Biotecnología, Universidad de Alicante, 03080 Alicante, Spain; laura.robles@ua.es (L.R.-G.); paula.saez@ua.es (P.S.-E.); elianamarina@gmail.com (E.M.L.-V.); andrea.lopbot25@gmail.com (A.L.-B.); 2 IVF Spain, Reproductive Medicine, 03540 Alicante, Spain; j.aizpurua@ivf-spain.com; 3 Cátedra Human Fertility, Universidad de Alicante, 03080 Alicante, Spain

**Keywords:** *Aleuria aurantia* lectin, capacitation, FE-SEM, fucose, glycoconjugates, sperm

## Abstract

The modification of sperm glycocalyx is an essential process during sperm capacitation. The presence and redistribution of terminal and linked fucose have been described during in vitro capacitation in humans. However, the influence of the capacitation time on the quantification and localization of terminal and linked fucose is still unknown. In this study, the quantitative and qualitative changes in fucosyl residues during different in vitro capacitation times (1 and 4 h), are simultaneously characterized by using *Aleuria aurantia* (AAA) lectin–gold labelling and high-resolution field emission scanning electron microscopy (FE-SEM) in human sperm. A significant decrease was found in the number of terminal fucose registered in the whole sperm head during the in vitro capacitation. Nevertheless, the quantification of fucose residues after 1 h of in vitro capacitation was very similar to those found after 4 h. Therefore, the changes observed in terminal and linked fucose during capacitation were not time-dependent. Furthermore, the comprehensive analysis of the topographic distribution showed the preferential fucosyl location in the acrosomal region and the presence of distinct clusters distributed over the head in all the studied conditions. Overall, these findings corroborate the validity of FE-SEM combined with gold labelling to register changes in surface molecules during in vitro sperm capacitation.

## 1. Introduction

The mammalian sperm glycocalyx is a 20–60 nm thick coat composed of a wide variety of glycocomponents, which play an important role in the transport, recognition, and interaction of gametes [1,2]. To date, the most convenient method for detecting glycoconjugate moieties has been the lectin assay [3,4]. This methodology revealed distinct changes in glycosylated sperm constituents during the capacitation process in mammals [5,6,7,8,9]. Thereby, the removal of sialic acid [10] or the remarkable mannose or N-acetylgalactosamine redistribution through different membrane domains has been reported during in vitro capacitation [5,9], elucidating the potential role of these conjugates.

In particular, fucosylated residues have been proposed to be involved in fertilization and embryonic development [11]. However, the (α-1,2) linked fucose characterization in mammalian sperm has presented several detection difficulties using fluorescence microscopy and *Ulex europaeus* (UEA) lectin [6,12]. On the other hand, the terminal (α-1,2) linked fucose and the (α-1,6) to N-acetylglucosamine or the (α-1,3) to N-acetyllactosamine linked fucose has been characterized by *Aleuria aurantia* (AAA) lectin in human sperm. Consequently, the AAA binding pattern in uncapacitated sperm has shown dotted fluorescence but labelled the acrosomal region in in vitro capacitated sperm [9,13]. Nevertheless, the quantitative changes and the topographical distribution of terminal and linked fucose residues during in vitro capacitation are not completely understood. In this context, the authors of this paper previously used field emission scanning electron microscopy (FE-SEM) to accurately localize and quantify mannose residues in human sperm surface during in vitro capacitation. Moreover, a significant and time-dependent decrease of mannose was registered during in vitro capacitation and a relocation mostly affecting acrosomal domain apical areas [14]. These findings showed the efficacy of FE-SEM and gold labelling as a useful tool for studying sperm surface components.

Considering the above, this paper showed both qualitative and quantitative changes in terminal and linked fucose residues during different times (1 and 4 h) of in vitro capacitation in human spermatozoa, by using FE-SEM technology.

## 2. Results

**Figure 1:**
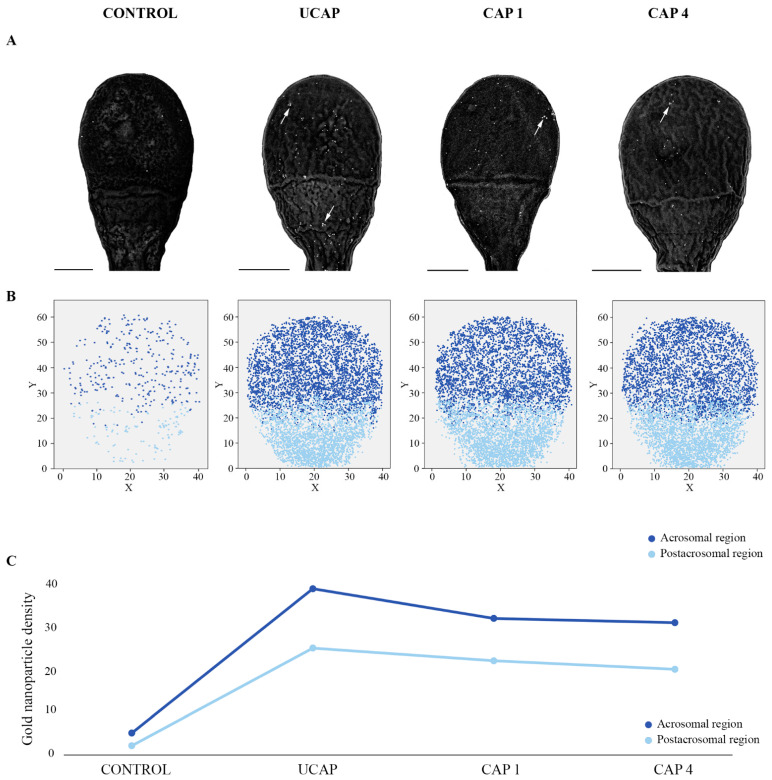
(**A**) Sperm FE-SEM micrographs of terminal and linked fucose recognized by AAA lectin in control by the inhibitory sugar test (CONTROL), uncapacitated (UCAP), one hour capacitated sperm (CAP1), and four hour capacitated sperm (CAP4). Note the presence of distinct clusters in all sperm conditions (white arrows). Scale bar of 1 µm common to all micrographs. (**B**) Topographical distribution maps resulting from the overlapping of fucosyl residues counts and position following a Cartesian coordinate system of 60 cells in control by the inhibitory sugar test and 105 cells for each UCAP, CAP1 and CAP4 conditions. (**C**) Trend graph showing the density after the incubation with the inhibitory sugar and the decrease in fucose residues in both acrosomal and postacrosomal regions during the in vitro capacitation time (1 and 4 h).

An inhibitory sugar test was performed to verify the specificity of binding to the residues recognized by AAA lectin and validate the method used in this study. Thereby, FE-SEM images and the topographical distribution map in control samples after the inhibitory sugar test showed a minimal presence of gold nanoparticles which were randomly distributed (Figure 1A,B). Moreover, the density recording in the presence of the inhibiting sugar evidenced the low quantity of gold nanoparticles. Specifically, an average of ~5 nanoparticles in the acrosomal and ~2 nanoparticles in the postacrosomal region (Figure 1C), were registered. Of note was the difference between the maximum and minimum gold nanoparticles number quantified in control samples, compared with UCAP, CAP1 and CAP4 conditions (Table 1).

The FE-SEM micrographs showed a higher concentration of terminal and linked fucose in the acrosomal region than in the postacrosomal region, in all the studied conditions (UCAP, CAP1, and CAP4) (Figure 1A). Moreover, researchers detected the presence of small circular areas with a high density of residues forming clusters both before and after in vitro sperm capacitation (white arrows Figure 1A). This finding could also be appreciated in the topographical distribution maps for each experimental condition (Figure 1B). Based on the topographical maps, the distribution of terminal and linked fucose in the sperm head in all conditions were quite similar, especially between CAP1 and CAP4 sperm (Figure 1B). However, a low-density distinct area in the center of the postacrosomal region principally in UCAP sperm was perceived, implying that the most of particles were in the periphery of this membrane domain (Figure 1B). 

The fucosyl density registration showed a significant decrease from ~65 residues in UCAP to ~50 in CAP1 and CAP4 in the whole sperm head (Kruskal–Wallis and Bonferroni *post-hoc* test, *p* < 0.05) (Table 1). In contrast, significant differences were not detected in the whole head density of fucose between CAP1 and CAP4 (*p* > 0.05). In the acrosomal region, a significant decrease was also found between UCAP and CAP1–CAP4 (*p* < 0.05) (Table 1, Figure 1C) but the differences between CAP1 and CAP4 in the fucose registered in the acrosomal region were not significant (*p* > 0.05). Regarding the fucosyl residues noted in the postacrosomal region, although the tendency was also decreasing during in vitro capacitation (Table 1, Figure 1C), the differences were not significant in any of the studied conditions (*p* > 0.05). On another hand, the acrosomal region showed a significantly higher fucose density than the postacrosomal region (Mann–Whitney U test, *p* < 0.01).

## 3. Discussion

The detection difficulties of fucosyl residues using conventional microscopy have required the development of more sensitive techniques [6,12]. In this study, field emission scanning electron microscopy (FE-SEM) allowed the researchers to accurately localize and quantify the terminal and linked fucose recognized by AAA lectin in human sperm surface during in vitro capacitation. Therefore, this study highlights the importance of high-resolution microscopy as a tool for the simultaneous qualitative and quantitative study of sperm surface molecules.

The results confirm the presence of the terminal and linked fucose in human sperm, agreeing with other results performed using lectins and fluorescence microscopy [13]. Moreover, the methodology described in this study allowed the quantification of fucose residues in both acrosomal and postacrosomal regions due to the high resolution offered by FE-SEM [15]. In this context, the nanoparticle density was found to be higher in the acrosomal than in the postacrosomal region both before and after in vitro capacitation. In accordance with this, previous reports have highlighted the role of fucose residues in human sperm as part of the recognition signal during fertilization [16,17,18]. This supports the majority presence of terminal fucose in the sperm anterior region of the head, which is the domain that allows interaction with the oocyte *zona pellucida* [19].

Additionally, a clear heterogeneity in the topographical distribution of fucosyl residues in sperm from the same physiological condition was noticed. In agreement, previous studies using AAA lectin and fluorescence microscopy suggested the presence of at least four different fluorescence patterns, confirming several and heterogeneous sperm populations in humans [9,13]. These authors further reported that the most common AAA binding pattern described in uncapacitated sperm (UCAP) showed distinct dotted fluorescence. Similarly, in the current study, several areas with high density of fucose arranged as clusters were observed in FE-SEM micrographs for UCAP. The majority presence of gold nanoparticles in the periphery of the postacrosomal region in UCAP was also recorded in this study, showing a very similar pattern to that previously described with fluorescence microscopy [13].

Studies performed with lectins and fluorescence microscopy allowed this study to characterize the qualitative changes in glycosylation. Unfortunately, fluorescence microscopy presents deficiencies in quantitative analysis [15]. As a novelty, the fucosyl residues present in human sperm surface were quantified using high-resolution microscopy (FE-SEM), registering a significant reduction of fucose after in vitro capacitation. Therefore, this technique provides noticeable advantages in comparison with the results obtained by fluorescence microscopy.

After in vitro capacitation by FE-SEM, a significant decrease in fucose residues located in the acrosomal region was registered. A previous study performed with fluorescence microscopy revealed a change in the AAA binding pattern after in vitro capacitation towards the acrosomal region [9]. This fucose redistribution during sperm capacitation could be due to the aggregation of lipidic microdomains in the anterior region of the sperm head. These domains contain a high concentration of proteins that interact with the *zona pellucida* of the oocyte. Since fucose residues are involved in the human recognition signal [16], this could explain the lipidic microdomain aggregation. In addition, these microdomains also contain the SNARE proteins responsible for the acrosome reaction [20].

The optimal time for in vitro capacitation is still a controversial issue and studies show contradictory results [21]. Previous research has reported different appropriate in vitro capacitation times depending on the lectin used in both humans and boars [9,22]. Thus, some authors propose better results with time-lasting protocols of more than 2 h [23], 3 or 4 h [24], or even a range of 3–24 h [25]. These outcomes suggest the differentiated response of the glycocalyx components to capacitation time. In this context, the authors of this current paper previously described a decrease in D-mannose residues during capacitation using the paired FE-SEM methodology [14]. Specifically, the density of D-mannose decreased significantly between the in vitro capacitated sperm during 1 (CAP1) and 4 h (CAP4), showing a clear time-dependent change. In the present study, unlike the results obtained for D-mannose, significant differences were not registered between the density of fucosyl residues in CAP1 and CAP4 sperm, being the topographic distribution highly similar in both experimental conditions. These results are in accordance with a previous study in which the same fucose majority pattern in CAP1 and CAP4 was described using fluorescence microscopy [9]. Therefore, this study’s data confirm that during sperm capacitation a redistribution of fucose residues occurs, but these changes are not time-dependent, since they do not require long capacitation times like other molecules.

## 4. Materials and Methods

### 4.1. Experimental Design

The experiment was approved by the Ethical Committee of the Universidad de Alicante (UA) according to the Declaration of Helsinki principles. Semen samples were obtained from three normozoospermic donors under informed written consent. Each sample was split into three experimental conditions as follows: uncapacitated spermatozoa (UCAP), one hour capacitated spermatozoa (CAP1), and four hour capacitated spermatozoa (CAP4). The one hour capacitation condition was chosen according to the World Health Organization (WHO) swim-up guidelines [26], and the four hour capacitation was based on previous studies [24]. Terminal and linked fucose disposition and quantification of human sperm glycocalyx was assessed in the three experimental conditions described above by FE-SEM.

### 4.2. Preparation of Semen Samples

Donor semen samples were obtained by masturbation, after three to five continuous days of sexual abstinence. The basic seminogram analysis was performed in the laboratory of the Departamento de Biotecnología at the UA following WHO guidelines [26]. Only normozoospermic samples were included in this study.

### 4.3. Sperm Capacitation

Sperm cells were capacitated by the swim-up technique in human tubal fluid medium (HTF, Origio^®^, Måløv, Germany) supplemented with 5 mg/mL of bovine serum albumin (BSA, Sigma-Aldrich^®^, Saint Louis, MI, USA) at 37 °C with 5% CO_2_ (*v*/*v*) during 1 (CAP1) and 4 h (CAP4). The supernatant, containing the motile cells (>95% of motility), was collected, and washed three times during 5 min in phosphate buffered saline (PBS, Biowest^®^, Nuaillé, France) by centrifugation (250× *g*, 10 min). Spermatozoa from the different experimental conditions (UCAP, CAP1 and CAP4) were fixed in 1% (*v*/*v*) glutaraldehyde (Electron Microscopy Sciences, Hatfield, PA, USA) for 45 min at 4 °C. Then, glutaraldehyde was replaced with PBS (PBS, Biowest, Nuaillé, France), pH 7.4, and the samples were stored at 4 °C.

### 4.4. Lectin Labeling

The AAA lectin was used due to the preference for fucose and terminal fucose residues on complex oligosaccharides and glycoconjugates [27,28]. The lectin labelling protocol was adapted from [14]. A total volume of 5 µL from each glutaraldehyde-fixed condition was placed on a round coverslip with a diameter of 12 mm. After being dried, and once cells were adhered, it was washed in distilled water (2 for 5 min) and in PBS (3 for 5 min). Then, it was incubated with a 2% BSA block solution for 30 min. Next to that, spermatozoa were subsequently incubated with AAA lectin peroxidase conjugate (*Aleuria aurantia* Lectin-HRP conjugate, Alpha Diagnostic International, San Antonio, TX, USA) at a final concentration of 1:100 for 1 h at room temperature in a humid chamber. Different AAA-HRP dilutions were tested to determine the optimal concentration. Afterwards, the sample was washed in PBS (3 for 5 min). Subsequently, the anti-peroxidase was added (Sigma-Aldrich, Saint Louis, MO, USA) (1:500) for 1 h and was washed again in PBS (3 for 5 min). After washing, the sample was incubated with protein A-colloidal gold conjugated (10 nm) (Cellular Biology Department, Utrech, Utrech University, The Netherlands) at a concentration of 1:70 for 1 h, and it was washed in PBS (3 for 5 min). A postfixation in 1% (*v*/*v*) glutaraldehyde was performed for 30 min. The sperm sample was washed in PBS (3 for 5 min) and distilled water (2 for 5 min). Finally, the dried simple was glued to the FE-SEM stubs with a carbon adhesive tape before being carbon sputter coated (SCD 004 Sputter Coater: Bal-Tec AG, Balzers, Liechtenstein). Simultaneously, an inhibitory sugar test was accomplished by treatment of the AAA lectin conjugate with 0.2 M of the corresponding L-fucose in order to corroborate the specificity of the lectin.

### 4.5. Field Emission Scanning Electron Microscopy Imaging

A total of 105 spermatozoa head digital micrographs (1024 × 768 pixels) were obtained at 18,000× magnification for each physiological condition. The calibration and recording of the density and topographical distribution of nanoparticles were previously described [14]. Briefly, calibrated micrographs were edited using Adobe Photoshop^®^ CS5 (Adobe Inc., San Jose, CA, USA) to obtain oriented images and the SigmaScan^®^ Pro (SPSSTM, Chicago, IL, USA) digital imaging software to count gold nanoparticles and to consider their topographic spatial distribution in both acrosomal and postacrosomal regions. The same procedure was performed for 20 randomly selected sperm head micrographs for each UCAP, CAP1 and CAP4 after the inhibitory sugar test. Consequently, the labelling method used in this study was evaluated.

### 4.6. Statistical Analysis

A Shapiro–Wilk (W) test was performed to test the distribution and equal variance in the gold nanoparticles counts, showing non-normal distribution (W = 0.9; *p* < 0.05). Thereafter, Kruskal–Wallis and Bonferroni *post-hoc* tests were conducted with the aim of determining differences in nanoparticle density on the different spermatozoa membrane domains and the physiological conditions included in this study (UCAP, CAP1, and CAP4). The Mann–Whitney U test was used to assess differences between gold nanoparticle densities between acrosomal and postacrosomal regions in the same condition. Descriptive and statistical results were obtained by means of IBM SPSS Statistics 19.0 (IBM, Armonk, NY, USA). Differences were statistically significant at a 95% confidence level (*p* < 0.05).

## 5. Conclusions

This study’s results showed novel changes in the terminal and linked fucose recognized by AAA lectin after in vitro capacitation. Concretely, FE-SEM microscopy showed a significant decrease in these sugar residues through the in vitro capacitation process. However, these changes were not capacitation time-dependent. Overall, to choose the most optimal capacitation time and improve sperm selection techniques, future research is necessary to increase knowledge about the physiological and molecular modifications that occur during sperm capacitation.

## Figures and Tables

**Table 1:** Densities of terminal and linked fucose recognized by AAA lectin registered in both the acrosomal and postacrosomal regions.

		Membrane Domain							
		Acrosomal Region				Postacrosomal Region			
Condition	n	Mean	SD	Min	Max	Mean	SD	Min	Max
CONTROL	60	5.23	2.30	1.00	9.00	1.95	1.52	0.00	6.00
UCAP	105	38.54	19.19	9.00	111.00	25.15	15.50	7.00	87.00
CAP1	105	31.84 ^a^	18.88	7.00	118.00	21.70	17.70	1.00	92.00
CAP4	105	30.50 ^b^	21.44	6.00	139.00	19.65	13.31	1.00	115.00

CONTROL: control sperm after the inhibitory sugar test; UCAP: uncapacitated sperm; CAP1: one hour capacitated sperm; CAP4: four hour capacitated sperm. Letters a and b indicate significant differences (*p* < 0.05) in fucose densities according to Kruskal–Wallis test and Bonferroni *post-hoc* between different physiological conditions: ^a^ significant difference between UCAP and CAP1, ^b^ significant differences between UCAP and CAP4.
